# Supported Poly(Ionic Liquid)-Heteropolyacid Based Materials for Heterogeneous Catalytic Fructose Dehydration in Aqueous Medium

**DOI:** 10.3390/molecules27154722

**Published:** 2022-07-23

**Authors:** Elisa I. García-López, Vincenzo Campisciano, Francesco Giacalone, Leonarda Francesca Liotta, Giuseppe Marcì

**Affiliations:** 1 Department of Biological, Chemical and Pharmaceutical Sciences and Technologies (STEBICEF), INSTM UdR—Palermo, University of Palermo, Viale delle Scienze, 90128 Palermo, Italy; elisaisabel.garcialopez@unipa.it (E.I.G.-L.); francesco.giacalone@unipa.it (F.G.); 2 Istituto per lo Studio dei Materiali Nanostrutturati (ISMN)-CNR, Via Ugo La Malfa 153, 90146 Palermo, Italy; leonardafrancesca.liotta@cnr.it; 3 “Schiavello-Grillone” Photocatalysis Group, Department of Engineering, University of Palermo, Viale delle Scienze, 90128 Palermo, Italy

**Keywords:** Hybrid materials, ionic liquids, heteropolyacid, fructose dehydration

## Abstract

Two sets of four different supported catalyst materials were prepared. One set was obtained by polymerization of a bis-vinylimidazolium salt, which formed a poly(ionic liquid) coating on SiO_2_, TiO_2_, boron nitride BN, and carbon nitride C_3_N_4_. The other set was, instead, obtained by immobilizing Keggin heteropolyacid H_3_PW_12_O_40_ onto poly-imidazolium functionalized materials. All the catalysts, including the bare supports, were subjected to physical and chemical characterization by XRD, SEM, Specific Surface Area and pore size measurements, TGA, FTIR, and acidity-basicity measurements. The catalytic activity of the materials was tested versus the fructose dehydration in water solution at two different sugar initial concentrations (0.3 and 1 M). Tests lasted 3 h with an amount of catalyst of 2 g∙L^−1^. The presence of the poly-imidazolium on the surface of the supports increased the catalytic conversion of fructose to 5-hydroxymethylfurfural (the most abundant compound obtained) and was further improved by the contemporary presence of the heteropolyacid, at least for the highest initial fructose concentration. In the latter conditions, the highest yield of 5-hydroxymethylfurfural (>40%) was also obtained.

## 1. Introduction

Biomass is a green and easily available renewable resource suitable as a source to obtain fuels or chemicals [1]. In this context, the valorization of hexoses, the most abundant monosaccharide present in biomass, represents a relevant issue. A variety of compounds can be obtained from hexoses and their catalytic dehydration in an acidic medium, giving rise to furanic compounds, and this is a way to obtain more added-value molecules. 5-Hydroxymethylfurfural (HMF) is obtained as the main product from the hexoses’ dehydration. HMF is a platform molecule, i.e., it can be used as a starting species to obtain other useful chemicals. By means of HMF hydrogenation, it is possible to obtain 2,5-dimethylfuran, a commonly used bioderived fuel, 2,5-bis(hydroxymethyl)furan, useful in the manufacture of polyurethane foam, or 2,5-dimethyltetrahydrofuran, used in polyester preparation [2] in the replacement of oil-derived chemicals for biofuel production and bio-polymeric plastic materials [3]. Furthermore, the hydration of HMF can yield levulinic acid, an important intermediate for polymer production [4,5]. Due to the great potential of HMF as a versatile platform molecule, its production has been largely investigated by several methodologies [6], among them fructose dehydration [7,8]. HMF production from fructose requires the removal of three water molecules; however, side products, particularly humins and/ or re-hydration and over-oxidation substances, can be obtained. Additionally, less dehydrated intermediate species can be formed, which in turn could also suffer inter-molecule condensation, producing polymers and insoluble humins. Despite the advantage of using water as an environmentally benign solvent, the chemistry in aqueous systems suffers from relatively low HMF yields because of subsequent reactions forming mainly insoluble humins. 

In general, dehydration reactions are achieved with strongly acidic catalysts such as H_2_SO_4_; however, the use of heterogeneous solid acid catalysts is by far more desirable for practical reasons. 

Heterogeneous catalysts would represent a valid solution since they are easily separated from the reaction mixture by filtration and therefore can be conveniently reused. Heteropolyacids (HPAs) have been extensively used as catalysts [9] and also in dehydration reactions due to their strong acidity and the Keggin H_3_PW_12_O_40_, hereafter labelled as PW_12_, is the most used by virtue of its high stability and acidity compared to other HPAs structures. In spite of their solubility in polar reaction media, heteropolyacids have been widely used as insoluble solid acid catalysts for liquid-phase reactions [10]. In recent years, HPA/ionic liquid hybrids have emerged as suitable catalytic systems in different processes such as hydroxylation of benzene [11], fixation of CO_2_ into epoxides [12] or selective oxidation of alcohols [13], among others. The dehydration of fructose is achieved under acidic conditions and HPAs are strong Brønsted acids that would reasonably replace inorganic or organic acids in this catalytic reaction. Moreover, ionic liquids (ILs) have been demonstrated to play significant roles in the conversion of fructose to 5-HMF as environmental-friendly green solvents. In this context, one very effective method, which has been selected in this study as a suitable synthetic route, is the direct grafting of PW_12_ to a supported poly ionic liquid-like crosslinked network supported by means of anion metathesis between bromide and H_2_PW_12_O_40_^−^ ions. The latter was generated by the reaction of phosphotungstic acid with one equivalent of NaHCO_3_. Indeed, the heterogeneous catalysts here prepared were composed of a support, namely SiO_2_, TiO_2_, C_3_N_4_ and BN where a poly ionic liquid-like network (IL) was grafted by means of radical polymerization of the corresponding bis-vinylimidazolium monomer (in the following named **imi**). Hence, the phosphotungstic heteropolyacid has been partially deprotonated and immobilized through anion exchange with bromide anions of the polymeric network. The catalytic behavior of these materials was then investigated for fructose dehydration in an aqueous medium to obtain HMF. Some preliminary experiments were performed to establish the optimal conditions to maximize HMF production from fructose using water as the solvent.

## 2. Results and Discussion

### 2.1. Preparation of the Materials

**Scheme 1:**
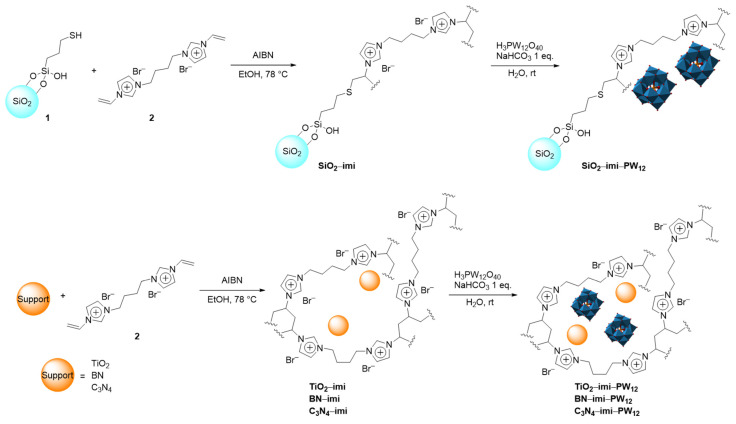
Synthetic procedure for the preparation of SiO_2_−imi−PW_12_, TiO_2_−imi−PW_12_, BN−imi−PW_12_, and C_3_N_4_−imi−PW_12_.

Four different support materials, namely SiO_2_, TiO_2_, boron nitride BN, and carbon nitride C_3_N_4_, were used to immobilize the bis-vinylimidazolium (**imi**) salt **2** (Scheme 1). Two strategies were employed for this purpose. In the case of SiO_2_ support, the **SiO_2_−imi** was prepared through an AIBN−initiated thiol–ene coupling between the mercaptopropyl-functionalized silica **1** and the bis-vinylimidazolium salt **2** obtaining **SiO_2_−imi** material (Scheme 1). Moreover, **TiO_2_−**, **BN−**, and **C_3_N_4_−imi** materials were obtained by means of the simple entrapment of the pristine support materials (TiO_2_, BN, and C_3_N_4_) inside the polymeric network formed after the AIBN−initiated radical polymerization of the bis-vinylimidazolium salt **2** (Scheme 1). Keggin heteropolyacid H_3_PW_12_O_40_ was eventually immobilized onto poly-imidazolium functionalized materials by means of ion exchange in water in the presence of one equivalent of NaHCO_3_ thus obtaining **SiO_2_−imi−PW_12_**, **TiO_2_−imi−PW_12_**, **BN−imi−PW_12_**, and **C_3_N_4_−imi−PW_12_** (Scheme 1).

### 2.2. Characterisation of the Samples

Some morphological properties of the prepared samples are listed in Table 1. The SSA values reflect those of the parent supports (reported in brackets in Table 1), although the functionalization with **imi** and **PW_12_** caused a decrease in the surface area and porosity. The huge decrease in SSA in the case of SiO_2_ functionalized material was already observed in a previous work [14] in which the SSA of a home prepared SiO_2_ sample (318 m^2^∙g^−1^) decreased up to (53 m^2^∙g^−1^) when the material was impregnated with PW_12_. In general, the observed decrease in the SSA for the supported catalysts can be explained by considering the partial blockage of the pores of the supports, due to the presence of **imi** and HPA clusters.

**Figure 1:**
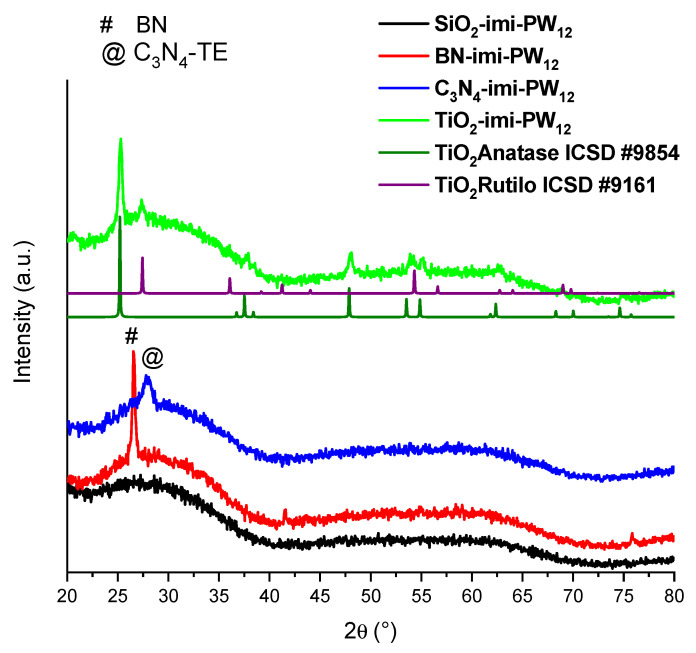
XRD patterns of the functionalized materials along with those of TiO_2_, both anatase and rutile.

In order to investigate the crystalline structure of the prepared materials, the XRD patterns were registered (Figure 1). The pattern of the **SiO_2_−imi−PW_12_** sample is amorphous, as the starting silica used. Peaks of the BN and C_3_N_4_ phases were detected in the diffractograms of **BN−imi−PW_12_** and **C_3_N_4_−imi−PW_12_**, respectively, while the **TiO_2_−imi−PW_12_** sample shows anatase and rutile features. It is important to note that none of the X-ray patterns of the functionalized materials exhibit the characteristic peaks of PW_12_. This fact can be explained by considering a good dispersion of the HPA on the support.

**Figure 2:**
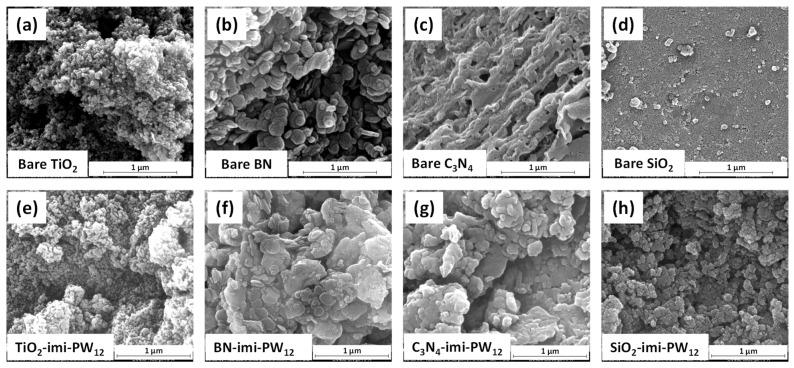
SEM picture of bare TiO_2_, BN, C_3_N_4_, and SiO_2_ supports and **TiO_2_−imi−PW_12_**, **BN−imi−PW_12_**, **C_3_N_4_−imi−PW_12_**, and **SiO_2_−imi−PW_12_** samples.

The SEM micrographs shown in Figure 2 are useful for studying the morphology of the samples investigated and especially for identifying any changes that may occur when the supports are loaded with the poly ionic liquid and PW_12_. From a comparison between bare the TiO_2_ and the **TiO_2_−imi−PW_12_** samples, it can be observed that both materials are constituted of agglomerates of particles which are smaller in the case of the bare support (ca. 20 nm) against ca. 80 nm measured for the supported material (Figure 2a,e). In the case of the BN based samples, it is possible to observe that the small sheets (up to ca. 200 nm) of boron nitride present in the pure BN are maintained in the supported sample even if in the latter case some of them appear fused each with other (size up to ca. 700 nm, Figure 2b,f). Instead, the **C_3_N_4_−imi−PW_12_** sample appears very different from the starting carbon nitride. In fact, in the former, it can be seen that there are only very few of the carbon nitride sheets observable in the photo of pure C_3_N_4_ (Figure 2c,g). It is likely that some of these sheets have been covered by PW_12_ and/or they have been broken due to an acid–base reaction with PW_12_ to form small pieces that finally agglomerated. In the case of the samples based on SiO_2_, the morphology of **SiO_2_−imi−PW_12_** appears slightly different with respect to that of the bare oxide. In particular, although the size of the particles of both the bare and supported silica are similar (ca. 50 nm), a new macroporosity appears in the latter. It is also possible in this case that the surface of the SiO_2_ has been completely covered by the poly ionic liquid and PW_12_ (Figure 2d,h).

**Figure 3:**
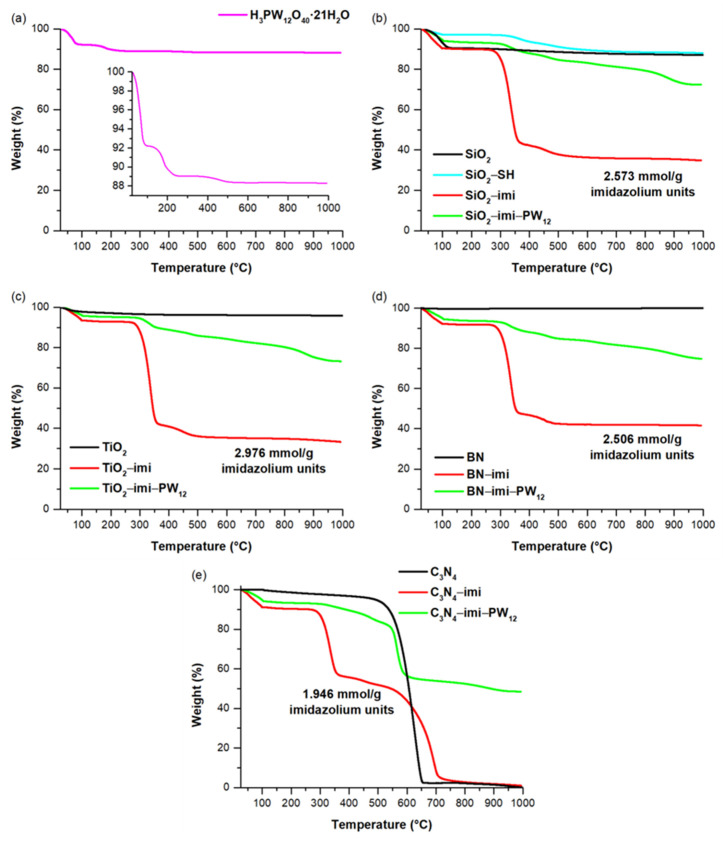
TGA under N_2_ of (**a**) H_3_PW_12_O_40_·21H_2_O, (**b**) **SiO_2_−imi** and **SiO_2_−imi−PW_12_**, (**c**) **TiO_2_−imi** and **TiO_2_−imi−PW_12_**, (**d**) **BN−imi** and **BN−imi−PW_12_**, and (**e**) **C_3_N_4_−imi** and **C_3_N_4_−imi−PW_12_**.

Keggin heteropolyacid H_3_PW_12_O_40_ and all the prepared materials were subjected to thermogravimetric analysis under nitrogen flow, as shown in Figure 3. The TGA of PW_12_, reported in Figure 3a, was in agreement with previous observations [15] with the first two decomposition processes at about 90 °C and 200 °C, corresponding to the loss of physisorbed and hydration water, respectively. Moreover, the prolonged mass loss starting at 400 °C leads to the final decomposition of the Keggin structure with the formation of WO_3_. TGA of all the polyimidazolium functionalized materials, before and after the PW_12_ immobilization, proved their high thermal stability, being as such hybrids, and were stable up to about 300 °C temperature, at which the degradation process of the poly-imidazolium layers starts (Figure 3b–e). The thermogravimetric analysis of pristine supports was also carried out. Pristine SiO_2_ showed a main weight loss between 25 and 120 °C due to the removal of physisorbed water on its surface, followed by a broader and less intense weight loss of 3.8 wt% between 120 and 1000 °C due to the condensation of OH groups (Figure 3b). A similar behavior was observed in the case of pristine TiO_2_, albeit with a lower weight loss of 1.8 wt% between 100 and 1000 °C (Figure 3c). The thermogravimetric profile of pristine BN support was constant during the whole temperature ramp from 25 to 1000 °C (Figure 3d), whereas C_3_N_4_ started to degrade at about 500 °C to give no residue at 1000 °C (Figure 3e). Therefore, the imidazolium loadings were estimated considering the weight loss between 130 and 1000 °C for **SiO_2_−imi** (2.573 mmol/g; loading determined by comparison of TG profiles of **SiO_2_−imi** and **SiO_2_−SH**—Figure 3b), **TiO_2_−imi** (2.976 mmol/g), and **BN−imi** (2.506 mmol/g) materials, whereas the 130–500 °C range was taken into account in the case of **C_3_N_4_−imi**. Furthermore, the total degradation of C_3_N_4_ support material at 1000 °C determined the PW_12_ loading in the case of **C_3_N_4_−imi−PW_12_**, by taking into account the residual weight at 1000 °C due to WO_3_ and corresponding to a PW_12_ loading of 0.175 mmol/g. Conversely, since the other support materials, namely SiO_2_, TiO_2_ and BN, are stable up to 1000 °C, the estimation of PW_12_ loading by examining the residual weights at this temperature is more difficult. Therefore, PW_12_ loadings of **SiO_2_−imi−PW_12_** (0.188 mmol/g), **TiO_2_−imi−PW_12_** (0.198 mmol/g), and **BN−imi−PW_12_** (0.182 mmol/g) were estimated by considering the experimental weight increase obtained after the immobilization of phosphotungstic acid, knowing that the supported species was H_2_PW_12_O_40_^−^.

FTIR analysis is a useful tool for the evaluation of the integrity of the heteropolyacid structure after the bonding to the imi deposited onto the supports. The Keggin anion (PW_12_) has four types of oxygen, which give four characteristic transitions in the range of 700–1200 cm^−1^. These bands have been assigned to the typical vibrations of Keggin unit at 1080 (stretching vibration (P-Oi)), 980 (stretching vibration of W=O), 890, and 810 cm^−1^ (corner and edge-sharing W–O–W vibrations) according to the literature data [16,17,18].

**Figure 4:**
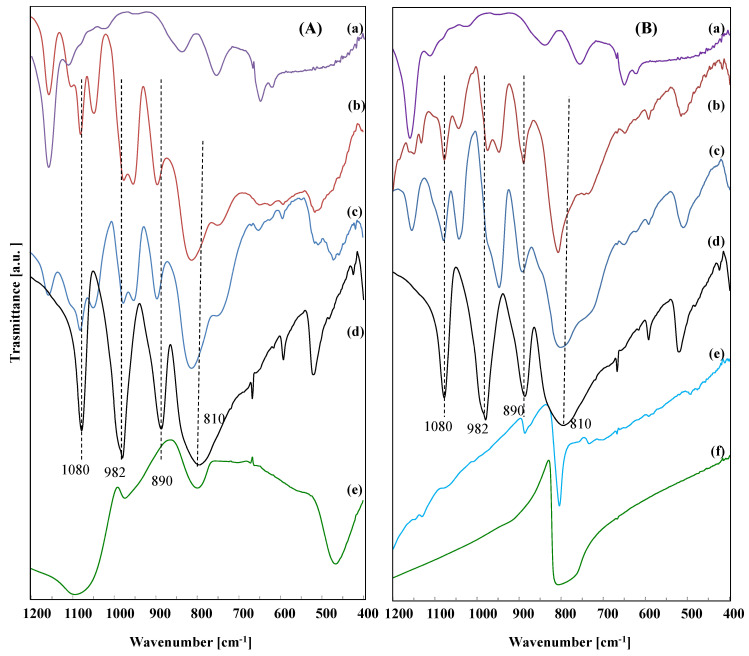
FTIR spectra of (**A**): (**a**) **imi**; (**b**) **TiO_2_−imi−PW_12_**; (**c**) **SiO_2_−imi−PW_12_**; (**d**) PW_12_ and (**e**) SiO_2_; and (**B**): (**a**) **imi**; (**b**) **C_3_N_4_−imi−PW_12_**; (**c**) **BN−imi−PW_12_**; (**d**) PW_12_; (**e**) C_3_N_4_ and (**f**) BN.

A perusal of Figure 4 reveals that there is no substantial shift in the position of the P–O stretching transition (1080 cm^−1^) or the W=O terminal oxygen one (980 cm^−1^); however, it is evident that there is a clear shift for the W–O–W stretching vibration frequencies in the prepared nanomaterials. The bands assigned to the edge-shared octahedral of the Keggin unit (W–Ob–W) and to the corner-shared octahedral of the Keggin unit (W–Oc–W) at 890 and 810 cm^−1^ in the pristine PW_12_ appear at higher wavenumbers in the support-imi−PW_12_ composites. This phenomenon is particularly clear for the **TiO_2_−imi−PW_12_** and **SiO_2_−imi−PW_12_** materials (see Figure 4A), but it is also evident for **C_3_N_4_−imi−PW_12_** and the **BN−imi−PW_12_** solid_._ The higher wavenumbers for the W–O–W stretching vibrations are consistent with strong interactions between the oxygens of the PW_12_ heteropolytungstate anion and imidazolium cation in the prepared nanomaterial, as observed before [19]. These frequency shifts indicate improved stability of the heteropolyacid in the material. An nteraction of the PW_12_ with the hydroxyls of the oxides supports, as reported in the literature for SiO_2_ [20] or TiO_2_ [21], cannot be excluded. Indeed, according to Rocchiccioli-Deltcheff et al., variations in the positions of the four characteristic IR transitions of the polyoxometalate could be explained by considering the effects of various interactions between the heteropolyanions and water molecules of crystallization [22]. The peaks corresponding to P-O and W=O vibration modes could be broadened or shifted with respect to the bare PW_12_ spectrum due to H-bonding interaction between the oxygen atom of the Keggin anion and hydroxyl groups. At the same time, the close maintenance of the vibrations at 1080 and 890 cm^−1^ reveals the preservation of the primary structure geometry of the Keggin cluster in the catalysts.

As mentioned in the introduction, it is well known that the dehydration of fructose is catalyzed in an acidic medium, consequently the acidity of the catalyst’s surface is an important parameter influencing the activity of the solids. The acidity of calcined materials is often determined using the temperature-programmed desorption of ammonia (NH_3_-TPD) [23]; however, it is not possible to carry out these measurements with the catalysts used in this research because the polyimidazolium network and also C_3_N_4_ decompose with temperature. Alternatively, Gervasini et al. have proposed the transformation of 2-propanol as a test reaction to study the acid–base character of the catalytic sites for oxides used as catalysts [24]. The dehydration of 2-propanol forming propene is catalyzed by an acid site, whereas the dehydrogenation to form propanone is catalyzed by both acid and basic sites through a concerted mechanism. Dehydration to propene is considered a good measure of the acidic properties of the catalyst surface. The catalytic 2-propanol dehydration forming propene by using supported Keggin heteropolyacid H_3_PW_12_O_40_ was already used to evaluate the acidity, of only Brønsted type, of oxides [23] and Keggin supported catalysts [25]. Preliminary experiments indicated that after an equilibration time (ca. 30 min. was enough) at room temperature in dark conditions, 2-propanol was partially adsorbed on the surface of all the catalysts used in this work. Under these conditions, the extent of 2-propanol adsorption was ca. 80% for all of the solids. After the adsorption/desorption equilibrium was reached, the catalytic experiments were started and carried out for 2 h at a temperature for which a catalytic activity towards the formation of propene and also propanone and diisopropyl ether was detectable, i.e., 120 °C. The reaction products were analyzed in the batch reactor at intervals of 30 min.

**Figure 5:**
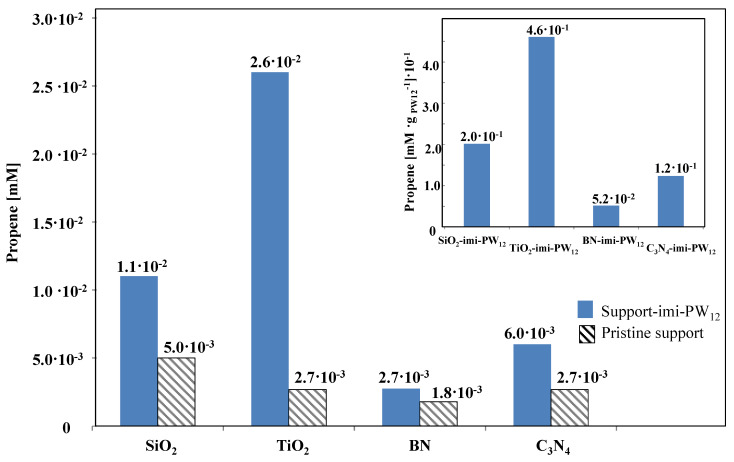
Propene amount measured in the atmosphere of the batch reactor after the 2-propanol catalytic dehydration, carried out for 120 min at 120 °C in the presence of 0.1 g of solid catalyst. (Inset) propene obtained per gram of PW_12_.

One important peculiarity of heteropolyacids is their ability to achieve a pseudo liquid phase inside their bulk [9]. This occurs in the spaces between the Keggin anion clusters of the crystalline structure where the acidic Brønsted sites, which represent the active sites for the catalytic reactions, are localized. Polar molecules, such as 2-propanol, are very soluble in the pseudo liquid phase and, consequently, it appears appropriate to follow the propene evolution [25], because it is not absorbed in the pseudo-liquid phase. It is worth mentioning that, in agreement with previous reports [23,24,25], propene appeared along with small amounts of propanone and diisopropyl ether, the formation of which was not strictly related to the Brønsted acidity. The amount of propene measured in the atmosphere of the reactor after the catalytic 2-propanol dehydration reaction is reported in Figure 5 both for the support-imi−PW_12_ samples and for the bare supports. From a perusal of Figure 5, it is evident that the amount of propene formed during the catalytic tests was lower in the case of bare supports with respect to the cases of support-imi−PW_12_ samples. This fact indicates that the presence of polyimidazolium network and heteropolyacid substantially increases the acidity of all the materials. The amount of heteropolyacid (PW_12_) present on each solid was slightly different, hence in the inset of Figure 5, the amount of propene obtained per gram of PW_12_ present in the corresponding solid was reported. It is noteworthy that the amount of propene obtained using 0.1 g of bare PW_12_ was 1 mM, the same as the initial substrate, indicating that, in this case, all the 2-propanol was selectively dehydrated to propene.

The results conclude that the solids with the highest content of acidic sites are those based on the oxides, particularly the TiO_2_ based material, seems to be the more acidic.

### 2.3. Catalytic Fructose Dehydration Activity

**Figure 6:**
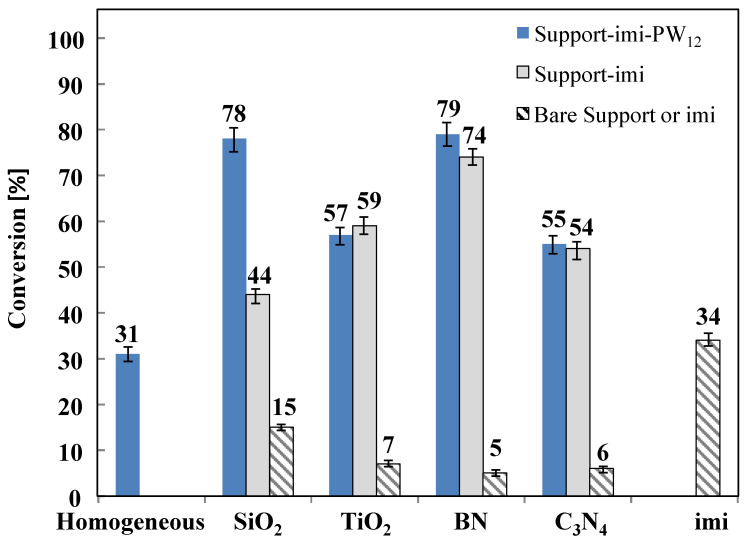
Conversion of fructose for catalytic reactions carried out in aqueous medium at 165 °C for 3 h in the presence of the different catalysts. Initial fructose concentration 0.3 M at natural pH.

**Figure 7:**
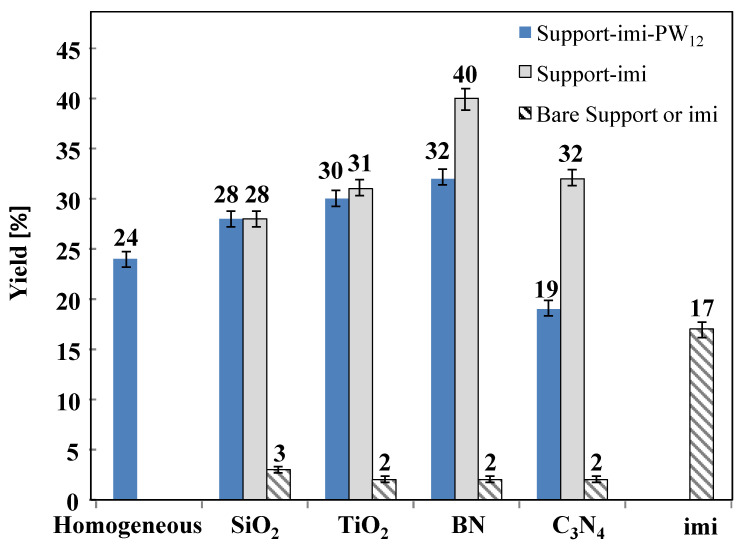
Yield of HMF for catalytic reactions carried out in aqueous medium at 165 °C for 3 h in the presence of the different catalysts. Initial substrate concentration 0.3 M at natural pH.

In a preliminary test carried out in homogeneous regime at natural pH (5.5) with an initial 0.3 M fructose concentration for a run at 165 °C lasting 3 h, the substrate conversion result was 31% whereas the selectivity to HMF was 79 and hence the yield in HMF ca. 24%. An analogous experiment carried out with a solution of fructose 1 M gave rise to a conversion of the substrate of 65% with a selectivity to HMF of 43, so a yield of 28% was obtained. The low yield to HMF can be explained by considering the formation of intermediates and their agglomeration/condensation side reactions to form insoluble polymers and humins. Indeed, in agreement with previous reports, we have observed that the reacting solution becomes a turbid and dark brown colored suspension after the catalytic experiments, due to the polymeric species formed. When the homogeneous catalytic reaction of 1 M fructose solution was carried out in the presence of a mineral acid (H_2_SO_4_ pH = 2.7) the conversion of the sugar was complete and the yield to HMF was 35%. The results of the heterogeneous catalytic fructose dehydration, in the presence of the various home prepared catalysts, in terms of fructose conversions are reported in Figure 6. It is worth mentioning that the values reported are the average of three measurements and the error could be at maximum a ± 7%, as indicated in all the figures by error bars. The pristine SiO_2_, TiO_2_, BN, and C_3_N_4_ are scarcely active in the reaction and the fructose conversion was lower than that obtained in homogeneous regime at natural pH, indicating that the presence of bare supports in the reaction medium inhibits the fructose dehydration. It is likely that in these cases most of the fructose disappearance is due to its adsorption onto the bare supports and not to its conversion. This fact will be clearer when the activity results, in terms of the yield to HMF, are discussed (see Figure 7).

The presence of the bare ionic liquid **imi** gives rise to an analogous activity compared to that obtained in homogeneous regime, indicating that the ionic liquid seems not able to affect the homogeneous reaction, at least when it was used alone in the reacting medium. On the contrary, the presence of the supported ionic liquid catalysts enhances the conversion of fructose in comparison to the experiments carried out in homogeneous system or in the presence of bare supports or the pristine **imi**. The addition of the Keggin heteropolyacid to the support-imi solid further increased the fructose conversion, (unless for the TiO_2_ based solid) particularly when SiO_2_ was used as support. The higher conversions were obtained in the presence of **BN−imi−PW_12_** and **SiO_2_−imi−PW_12_** composite.

Among the solids lacking in heteropolyacid, the use of the **BN−imi** catalyst gave rise to the best conversion (74%), slightly lower than that observed for the **BN−imi−PW_12_**. On the contrary, for **SiO_2_−imi**, a dramatic decrease in fructose conversion with respect to **SiO_2_−imi−PW_12_** was observed (from 78 to 44%). The differences between the fructose conversion obtained by using **TiO_2_−imi** and **C_3_N_4_−imi** both in the presence and in the absence of PW_12_ were modest, and the presence of heteropolyacid that resulted is almost irrelevant. Indeed, for **TiO_2_−imi** and **C_3_N_4_−imi,** very similar fructose conversions were obtained in comparison with the analogous solids containing PW_12_.

In summarizing, it is interesting to observe that by comparing the tests carried out in the presence of the bare supports to those performed in the presence of the support-imi−PW_12_ catalysts, the last ones appear more active in the conversion of fructose. This increase in activity is undoubtedly due to the higher acidity observed (see Figure 5) for the support-imi−PW_12_ materials with respect to those of the bare supports. However, careful observation of the results reported in Figure 6 shows that the conversion of fructose in the presence of **BN−imi−PW_12_**, which is the least acidic catalyst (see Figure 5), is the highest in absolute. Furthermore, the activity of the **C_3_N_4_−imi−PW_12_** sample, one of the least acidic catalysts, is similar to that of **TiO_2_−imi−PW_12_**, which is the material with the highest acidity. These results suggest that the acidity of the catalyst is not the only parameter influencing its activity. Moreover, we must keep in mind that the acidity measurements of the materials were carried out on the powder and not in aqueous suspension, which is the condition used in the catalytic tests. Consequently, water can play an important role as a function of its different interactions with the various materials and, in certain cases, it can have a levelling effect on the catalytic activity towards the transformation of fructose.

The yields to HMF for the same experiments reported in Figure 6 are depicted in Figure 7 and a perusal of these results allows one to conclude that the presence of PW_12_, in these conditions, resulted in irrelevant and in some cases slightly detrimental (**BN−imi−PW_12_** and **C_3_N_4_−imi−PW_12_** composite) at least when the initial fructose concentration was 0.3 M. It is noteworthy that the yield to HMF was worst in the presence of the pristine ionic liquid (**imi**) than that obtained in the homogeneous reaction. This fact indicates a lower selectivity (the conversion was almost the same) of bare **imi** versus the HMF formation with respect to the homogeneous reaction carried out at natural pH.

The **BN−imi−PW_12_** and **BN−imi** catalysts give rise to the best yield, particularly in the absence of the heteropolyacid (40%), whereas **SiO_2_−imi**, **TiO_2_−imi**, and **C_3_N_4_−imi** powders gave rise to a yield of ca. 30%. No difference in the HMF yield was observed between the solid in the presence or the absence of heteropolyacid when the oxides, SiO_2_ or TiO_2_, were used as supports. On the contrary, the presence of heteropolyacid reduces the selectivity and consequently the yield to HMF of the composites containing the 2D materials (BN and C_3_N_4_) as support.

**Figure 8:**
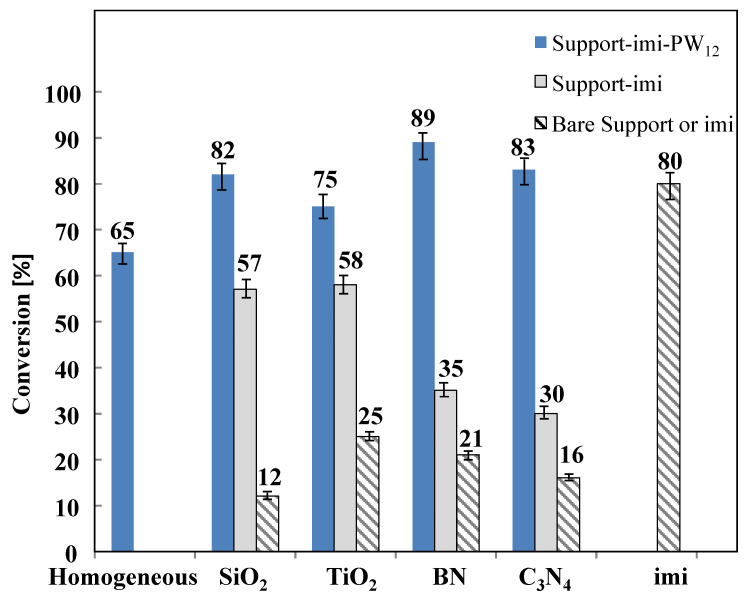
Conversion of fructose for catalytic reactions carried out in aqueous medium at 165 °C for 3 h in the presence of the different catalysts. Initial substrate concentration 1 M at natural pH.

As far as the yield obtained in the presence of the bare supports is concerned, the obtained values were very low (2–3%). This fact strengthens the hypothesis that pure supports inhibit the conversion of fructose to HMF and that the decrease in the concentration of the substrate is essentially due to its adsorption on the support surfaces. In order to study the influence of the fructose concentration on its conversion to HMF, a further set of experiments was performed with a higher initial concentration of fructose (1 M), and the obtained results are reported in Figure 8 in terms of sugar conversion. In general, both in homogeneous and heterogeneous regimes, the higher the initial fructose concentration the higher the amount of sugar converted. Indeed, some of us have before observed a higher fructose conversion when the initial concentration was increased from 0.3 M to 1 M both by using Nb_2_O_5_ based catalysts [21] and in the presence of sulfonated titania [26,27] for the same reaction and experimental conditions. 

From a perusal of Figure 8, the conversion of fructose was higher in the presence of ionic liquid (imi) and support-imi−PW_12_ in comparison to that obtained in homogeneous regime. The fructose conversion was much higher when the heteropolyacid was present on the solid with respect to the support-imi material. The higher conversions were obtained in the presence of the **BN−imi−PW_12_**, **C_3_N_4_−imi−PW_12_,** and **SiO_2_−imi−PW_12_** catalysts. The materials composed of support-imi attained the same conversion for the composites in which the oxides were used as supports, **SiO_2_−imi** and **TiO_2_−imi**, reaching 57–58%, whereas the materials containing the 2D materials as supports gave rise to almost the same conversion of around 30–35%. The conversion of fructose in the presence of the bare supports was very low, indicating that the disappearance of fructose is, also in this case, at least in part due to its adsorption on the surface of the catalyst.

**Figure 9:**
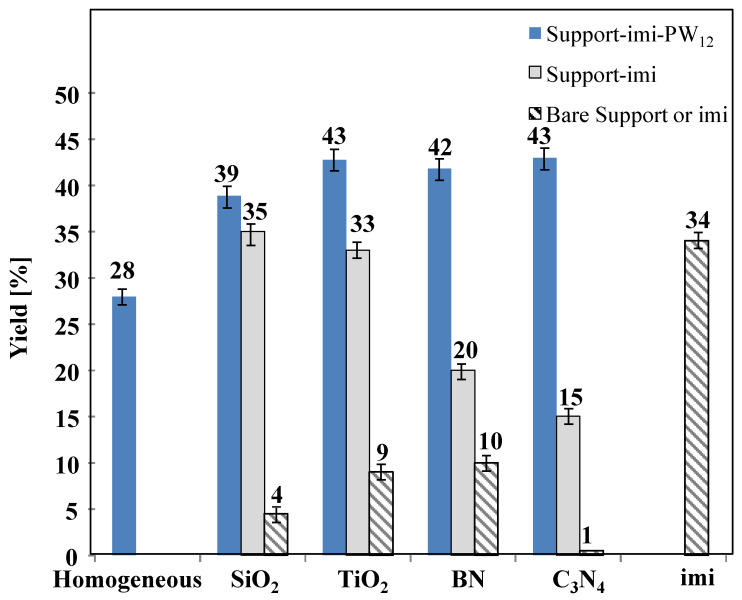
Yield of HMF for catalytic reactions carried out in aqueous medium at 165 °C for 3 h in the presence of the different catalysts showing the different supports. Initial substrate concentration 1 M at natural pH.

As far as the yields to HMF are concerned, Figure 9 reports the results obtained for the runs carried out with a fructose initial concentration equal to 1 M. Contrary to what was observed in the case of tests carried out starting from 0.3 M fructose solutions, in the case of 1 M sugar initial concentration, the yields to HMF were higher for materials containing PW_12_ and they resulted somewhat flatten for all the samples, attaining in any case, a value of ca. 40%. Consequently, the presence of heteropolyacid appears to be beneficial for obtaining HMF. Interestingly, the yield to HMF was ca. 33–35% for **SiO_2_−imi** and **TiO_2_−imi** and ca. 15–20% for the catalysts with the 2D support, **BN−imi** and **C_3_N_4_−imi**, but when PW_12_ was added the yield increased for all the catalysts up to ca. 40%. 

From the analysis of the obtained results, we can conclude that an increase in the initial concentration of fructose gives rise to an increase in the HMF yield, at least in the case of composites containing PW_12_. The increase in the yield is essentially due to the increased percentage of fructose converted. In fact, the selectivity towards the formation of HMF seems less affected by the increase in the initial concentration of fructose. On the contrary, in the case of the tests carried out in the presence of the **BN−imi** and **C_3_N_4_−imi** catalysts, the increase in the initial concentration of fructose causes a decrease in the yield of HMF, which is essentially due to the reduced percentage of sugar converted. In fact, although for all catalysts the absolute amount of sugar converted increases by increasing the initial concentration of fructose, its percentage of conversion does not follow the same trend for all of them and, in the case of the **BN−imi** and **C_3_N_4_−imi** catalysts, a lower percentage of sugar conversion was observed when the fructose initial concentration was higher.

In all cases, the dark solid residues obtained after the catalytic experiments were recovered and dried at 60 °C overnight. The brownish-orange color was much more intense for the experiments carried out with a fructose initial concentration of 1 M than for those using 0.3 M, indicating the higher formation of undesired species in the former case. The complex nature of this material that resulted in polymeric humins makes their characterization difficult; however, some specific FTIR bands, in agreement with literature findings, account for their presence where the formation of cross-linked furan rings via intermolecular dehydration derived from HMF occurs [28,29,30].

By comparing the results obtained in this work with those reported in the literature in analogous catalytic experiments devoted to the dehydration of fructose water solutions, it can be concluded that the maximum yield of HMF here reached (43%, with a fructose conversion of 83%) is a fairly good result. Indeed, for instance, some of us have previously obtained, by using the same set-up in the current investigation but employing Nb_2_O_5_ as heterogeneous catalyst, a yield to HMF of ca. 57% with a fructose conversion of ca. 80%. In this case, the initial fructose concentration was 1 M and the catalyst amount used was 1 g∙L^−1^ and the dehydration reaction of fructose was carried out at 165 °C [21]. Whereas Stosic et al. reported, also in the presence of Nb_2_O_5_, a conversion of fructose of ca. 87% with a yield to HMF of 36% at 130 °C starting from a 0.056 M aqueous solution of fructose in the presence of 1.33 g∙L^−1^ of catalyst [31]. Testa et al. have, instead, obtained at 165 °C an HMF yield of ca. 50% with a total conversion of fructose starting from an aqueous solution of 1.1 M in the presence of TiO_2_−SO_3_H (2 g∙L^−1^) as the catalyst [26].

## 3. Experimental

### 3.1. Preparation of the Catalysts

All chemicals were from Sigma Aldrich (Milano, Italy) and Strem Chemicals Inc. (Newburyport, MA, USA) with assay of 99.99% and used as received without any further purification. Water used throughout the experiments was purified by means of a Milli-Q system. The 1,4-butanediyl-3,3′-bis-1-vinylimidazolium dibromide has been prepared accordingly to the reported procedure already published by some of us [32].

As far as the supports are concerned, TiO_2_ was received from Evonik (TiO_2_ Evonik P25; Essen, Germany), BN and SiO_2_ from Sigma Aldrich. The first two materials were used as received and, on the contrary, SiO_2_ was thiol-functionalized as reported in our previous study [33]. C_3_N_4_ was prepared in a two-stage process, i.e., a thermal condensation of melamine (Sigma Aldrich) followed by a thermal exfoliation step [34].

#### 3.1.1. Preparation of Supported Poly-Imidazolium Bromide (SiO_2_−imi, TiO_2_−imi, BN−imi and C_3_N_4_−imi)

**SiO_2_−**In a two-neck round bottom flask, **1** (300 mg, 1.22 mmol/g SH) was dispersed in a solution of bis-vinylimidazolium salt **2** (600 mg, 1.48 mmol) in ethanol (10 mL) in the presence of azobisisobutyronitrile (AIBN; 24.60 mg, 0.15 mmol). After bubbling the reaction mixture with argon for 15 min, the flask was immersed into a preheated oil bath at 80 °C and refluxed at this temperature, under an argon atmosphere, for 16 h. The solid was filtered through a PTFE filter (JHWP; 0.45 μm) and washed with hot methanol and diethyl ether before drying under vacuum. **SiO_2_−imi** was obtained as a white powder (668 mg). Moreover, **TiO_2_−**, **BN−**, and **C_3_N_4_−imi** materials were obtained using simple entrapment of the pristine support materials (TiO_2_, BN, and C_3_N_4_; 300 mg) inside the polymeric network formed after the AIBN−initiated (24.60 mg, 0.15 mmol) radical polymerization of the bis-vinylimidazolium salt **2** (600 mg, 1.48 mmol) according to the general procedure reported for **SiO_2_−imi**. The following amounts of functionalized materials were obtained: **TiO_2_−imi** (852 mg), **BN−imi** (694 mg), and **C_3_N_4_−imi** (575 mg).

#### 3.1.2. Preparation of HPA Based Materials (SiO_2_−imi−PW_12_, TiO_2_−imi−PW_12_, BN−imi−PW_12_, and C_3_N_4_−imi−PW_12_)

Keggin heteropolyacid H_3_PW_12_O_40_ was eventually immobilized onto poly-imidazolium functionalized materials by means of ion exchange in water in the presence of one equivalent of NaHCO_3_ thus obtaining **SiO_2_−imi−PW_12_** (1.088 g), **TiO_2_−imi−PW_12_** (1.165 g), **BN−imi−PW_12_** (1.052 g), and **C_3_N_4_−imi−PW_12_** (1.002 g) (Scheme 1).

In detail, in a 10 mL round bottom flask, 500 mg of poly-imidazolium functionalized material (SiO_2_−, TiO_2_−, BN−, or C_3_N_4_−imi) was dispersed using sonication (20 min) in a solution of H_3_PW_12_O_40_∙21H_2_O (1 g, 0.307 mmol) in water (2 mL) before adding an aqueous solution (2 mL) of NaHCO_3_ (25.79 mg, 0.307 mmol). The mixture was stirred at room temperature for 21 h and then filtered through a PTFE filter (JHWP; 0.45 μm) and washed with water, methanol, and diethyl ether. The materials obtained were dried under vacuum at 50 °C for 1 h.

### 3.2. Characterization of the Catalysts

The crystalline structure of the samples was determined using powder X-ray diffraction patterns (XRD). Characterizations were carried out on a Bruker D5000 diffractometer equipped with Kristalloflex 760 X-ray generator (Bruker AXS GmbH, Karlsruhe, Germany) and with a curved graphite monochromator using Cu Kα radiation (40 kV/30 mA). The data were recorded in a 2θ range of 20°–80° with a step size of 0.05° and time per step of 20 s. The crystalline phases of samples were analyzed according to ICSD (Inorganic Crystal Structure Database) files.

Scanning electron microscopy (SEM) was performed using an FEI Quanta 200 ESEM microscope, operating at 20 kV on specimens upon which a thin layer of gold had been evaporated. The specific surface areas (SSA), pore volume and pore size of the materials were measured by N_2_ adsorption–desorption isotherms using a Micromeritics ASAP2020 system (Micromeritics Instrument Corp., Norcross, GA, USA). Before analysis, the samples were degassed in vacuum at 250 °C for 2 h, then the measurement was recorded at liquid nitrogen temperature (−196 °C). The Brunauer–Emmett–Teller (BET) method was used to calculate the SSA. The Barrett–Joyner–Halenda (BJH) method was applied to the desorption branch to estimate the pore volume and pore size distribution.

Thermogravimetric analyses (TGA) were registered under a nitrogen flow from rt to 1000 °C with a heating rate of 10 °C/min with a Mettler Toledo TGA/DSC STAR System. The temperature was raised from room temperature up to 100 °C, and then this temperature was maintained for 30 min to remove adsorbed water before reaching 1000 °C. Infrared spectra of the used samples were performed in KBr (Aldrich) pellets and were obtained with an FTIR-8400 Shimadzu spectrophotometer and recorded with 4 cm^−1^ resolution and 256 scans. 

According to Gervasini et al., the transformation of isopropanol is a test reaction suitable to study the acid–base character of the catalytic sites of the oxides [24]. To that aim, the catalytic transformation of isopropanol was carried out in a 25 mL cylindrical Pyrex batch reactor provided with a silicon/teflon septum containing 0.1 g of catalyst and where N_2_ was fluxed for ca. 0.5 h. The liquid isopropanol (2 μL) was introduced and vaporized in the reactor (Co = 1 mM), which was maintained at room temperature in dark conditions to achieve the adsorption equilibrium of the substrate on the catalyst surface. The reactor was successively heated up to 120 °C in a static oven. The evolution of the formed organic products was followed by performing withdrawals with a gas-tight syringe and injecting the vapor in a GC-2010 Shimadzu equipped with a Phenomenex Zebron Wax-plus column and an FID.

### 3.3. Catalytic Fructose Dehydration Experiments 

The fructose dehydration reaction was carried out in a 25 mL Teflon covered beaker placed in a stainless-steel autoclave heated at 165 °C. The reactor contained 6 mL of 0.3 or 1 M fructose aqueous solution. The amount of catalyst was 2 g∙L^−^^1^ and the reaction lasted 3 h. In order to identify and quantify all the compounds in the reaction mixture, the solution was analyzed by injection on Thermo Scientific Dionex Ultimate 3000 HPLC equipped with a Diode Array and refractive index detectors. The column was a REZEK ROA Organic acid H^+^ Phenomenex and the eluent was an aqueous 2.5 mM H_2_SO_4_ solution with a flow rate equal to 0.6 mL min^−1^. Retention times and UV spectra of the compounds were compared with those of standards purchased from Sigma-Aldrich with a purity of >99%. The catalytic performance in terms of conversion of fructose (X), selectivity towards HMF formation (S), and HMF yield (Y) was defined as follows:

(1) $$X=\frac{{\left[\mathrm{fructose}\right]}_{i}-{\left[\mathrm{fructose}\right]}_{f}}{{\left[\mathrm{fructose}\right]}_{i}}\times 100$$

(2) $$S=\frac{\left[\mathrm{HMF}\right]}{{\left[\mathrm{fructose}\right]}_{i}-{\left[\mathrm{fructose}\right]}_{f}}\times 100$$

(3) $$Y=\frac{\left[\mathrm{HMF}\right]}{{\left[\mathrm{fructose}\right]}_{i}}\times 100$$

where [fructose]_i_ and [fructose]_f_ are the initial and the final (after 3 h of reaction) concentrations of fructose, respectively, and [HMF] is the concentration of HMF at the end of the catalytic test.

## 4. Conclusions

In the present work, two sets of catalysts for the dehydration of fructose in water solution were prepared. The materials were obtained by supporting a bis-vinylimidazolium bromide salt on SiO_2_, TiO_2_, boron nitride BN, and carbon nitride C_3_N_4_ and by immobilizing Keggin heteropolyacid H_3_PW_12_O_40_ onto poly-imidazolium functionalized materials. The experiments to test the catalytic activity of the materials (2 g∙L^−1^) were performed at 165 °C at two different sugar initial concentrations (0.3 and 1 M) and lasted 3 h. 

The presence of the bare supports in the reacting medium inhibited the homogeneous reaction of fructose dehydration and, in these cases, most of the fructose disappearance is probably due to its adsorption onto the supports. The presence of the poly-imidazolium on the surface of the supports increased the catalytic conversion of fructose to 5-hydroxymethylfurfural (the most abundant compound obtained). The activity of the catalysts was further improved by the contemporary presence of the heteropolyacid, at least for the highest initial fructose concentration. In the latter conditions, the highest yield of 5-hydroxymethylfurfural (>40%) was also obtained. 

## Figures and Tables

**Table 1:** Specific Surface Area (SSA), cumulative pore volume and pore size of the functionalized materials.

Sample	SSA (m^2^∙g^−1^) ^a^	BJH Cumulative Pore Volume (Desorption Branch) (cm^3^∙g^−1^)	BJH Pore Size (Desorption Branch) (nm)
**SiO_2_−imi−PW_12_**	98 (400 SiO_2_)	0.20	6.95
**TiO_2_−imi−PW_12_**	53 (55 TiO_2_)	0.13	8.0
**BN−imi−PW_12_**	15 (21 BN)	0.07	17.8
**C_3_N_4_−imi−PW_12_**	62 (65 C_3_N_4_)	0.24	15.6

^a^ In brackets are the reported SSA values of the bare supports.

## Data Availability

The data presented in this study are available on request from the corresponding author.
